# The Finnish Biodiversity Information Facility as a best-practice model for biodiversity data infrastructures

**DOI:** 10.1038/s41597-021-00919-6

**Published:** 2021-05-25

**Authors:** Leif Schulman, Kari Lahti, Esko Piirainen, Mikko Heikkinen, Olli Raitio, Aino Juslén

**Affiliations:** 1 Biodiversity Informatics Unit, Finnish Museum of Natural History ’Luomus’, University of Helsinki, P.O. Box 17, FI-00014 Helsinki, Finland; 2 Present Address: Finnish Environment Institute SYKE, Latokartanonkaari 11, FI-00790 Helsinki, Finland; 3 Zoology Unit, Finnish Museum of Natural History ’Luomus’, University of Helsinki, P.O. Box 17, FI-00014 Helsinki, Finland

**Keywords:** Climate-change ecology, Biodiversity, Biogeography, Ecological modelling, Taxonomy

## Abstract

Biodiversity informatics has advanced rapidly with the maturation of major biodiversity data infrastructures (BDDIs), such as the Global Biodiversity Information Facility sharing unprecedented data volumes. Nevertheless, taxonomic, temporal and spatial data coverage remains unsatisfactory. With an increasing data need, the global BDDIs require continuous inflow from local data mobilisation, and national BDDIs are being developed around the world. The global BDDIs are specialised in certain data types or data life cycle stages which, despite possible merits, renders the BDDI landscape fragmented and complex. That this often is repeated at the national level creates counterproductive redundancy, complicates user services, and frustrates funders. Here, we present the Finnish Biodiversity Information Facility (FinBIF) as a model of an all-inclusive BDDI. It integrates relevant data types and phases of the data life cycle, manages them under one IT architecture, and distributes the data through one service portal under one brand. FinBIF has experienced diverse funder engagement and rapid user uptake. Therefore, we suggest the integrated and inclusive approach be adopted in national BDDI development.

## Introduction

Biodiversity informatics – the application of informatics techniques and technologies to collate, harmonise, manage, share, and use data and information on the world’s biota – has progressed considerably in the last two decades^1^. While the first proposals akin to biodiversity informatics date from the 1950s^2^, the development towards large-scale international initiatives started mostly in the late 1990s. The result was the initiation of global data resources such as the Catalogue of Life (CoL; a global index of known species) and the Global Biodiversity Information Facility (GBIF; an aggregator and distributor of species occurrence records), both established in 2001. More recently, initiatives sourcing and distributing new data types, such as the International Barcode of Life (iBOL; a DNA barcode reference library, 2008) and iNaturalist (a network and app for recording, sharing and identifying species observations, 2011) have emerged. Industrial-scale efforts to digitise natural history collections and the data they harbour have also taken off, e.g., Advancing Digitization of Biodiversity Collections (ADBC) in the USA and e-ReColNat in France.

These developments have made unprecedented volumes of data readily available for end-users. GBIF now offers > 1.6 billion occurrence records (gbif.org; 26 January 2021) and iBOL’s library covers 320,000 species (boldsystems.org; 26 January 2021). Nevertheless, the taxonomic, temporal and spatial coverage of the available data is still far from complete. For instance, of the c. 1.25 million described multicellular species, 0.8% are birds and 3.5% are fungi^3^, whereas in the GBIF-mediated records their shares are 62% and 1.3%, respectively. Similarly, 9.6 million GBIF records predate the year 1900, 353 million are from the first and 273 million from the second half of the 20^th^ century, and 1.2 billion from the first fifth of the 21^st^ century. Outside North America, Europe and Australasia, terrestrial areas are sparsely covered, and most of the oceans are void of data. The underrepresented areas tend to lack national data aggregators^1^.

The biodiversity crisis is accelerating and data gaps that hinder tackling it have been identified^4^. Thus, efforts to source and mobilise more biodiversity data should continue with an emphasis on reducing content biases. Apart from observational data platforms receiving their input from private citizens, the business model of the international biodiversity data infrastructures (BDDIs) includes heavy reliance on networks of participating national nodes or institutions for data inflow. Several national BDDIs already exist, but since many more are currently being developed around the world, seeking best-practice models is timely. Success in responding to the growing need of biodiversity data hinges on how efficiently data are mobilised in national initiatives.

**Fig. 1:**
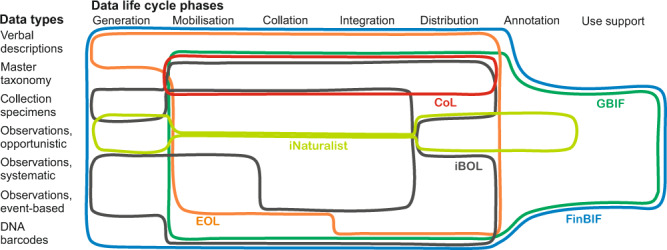
Service models of selected international biodiversity data infrastructures (BDDIs) and FinBIF. The model is depicted as a combination of data types supported and life cycle phases covered; *generation* = creating digital data through digitising analogue data or capturing born-digital data; *mobilisation* = enabling pre-existing digital data to become publicly findable and accessible; *collation* = gathering independent datasets to one repository; *integration* = making separate datasets an interoperable data mass; *distribution* = enabling data browsing, search, download and reuse; *annotation* = enabling crowd-sourced and expert improvement and enrichment of data; *use support* = providing dashboard and/or e-Lab functions for data visualisation and analysis. CoL = Catalogue of Life; EOL = Encyclopedia of Life; GBIF = Global Biodiversity Information Facility; iBOL = International Barcode of Life.

The global BDDIs tend to have specialised niches representing only a narrow segment of the multidimensional biodiversity informatics space^5^. They differ by data type(s) handled and data life cycle phase(s) supported. They may deal with only one data type (e.g., eBird, a platform for bird observations) or support one or a few links in the data mobilisation chain (e.g., GBIF that collates, integrates and distributes digital data but does not digitise analogue data; see Fig. 1, Table 1). The worldwide biodiversity informatics landscape is therefore composed of numerous elements, which have invested much effort in connecting to each other to provide a complete service array to end-users but have not always succeeded in avoiding redundancy^5^.

**Table 1:** Comparative information of a worldwide selection of biodiversity data infrastructures (BDDIs), FinBIF highlighted in bold.

BDDI	Data sourcing	Content coverage	# of records	# of spp.	Recs / capita	A	B	C	D	E	F	G	1	2	3	4	5	6	7	8
GBIF	Global	global; all	1.40 G	1.09 M		−	+	+	+	+	+	+	−	−	+	−	+	+	−	+
EOL	Global	global; all	13.9 M	2.05 M		+	+	+	+	+	−	+	−	−	+	−	−	+	−	−
iBOL	Global	global; all	7.54 M	0.30 M		−	+	+	−	+	+	+	+	−	+	−	−	−	−	+
iNaturalist	Global	global; all	23.8 M	0.22 M		−	+	−	+	−	−	−	−	+	+	−	+	+	−	−
Antmaps	Global	global; ants	2.41 M	15.5 k		−	+	+	+	−	−	−	+	−	+	−	+	−	−	−
FishBase	Global	global; fishes	1.00 M	34.3 k		+	+	+	+	+	−	−	−	+	+	−	+	+	−	−
ALA	Australia	global; all	86.8 M	0.26 M	3.47	+	+	+	+	+	+	+	+	+	+	−	+	+	−	+
SiBBr	Brazil	ntnl; all	15.6 M	0.22 M	0.07	−	+	+	+	+	+	+	−	−	+	−	0	−	−	0
eElurikkus	Estonia	global; all	4.50 M	0.11 M	3.41	+	+	+	+	+	+	+	+	+	+	+	+	+	−	+
**FinBIF**	**Finland**	**global; all**	**35.3** **M**	**38.0 k**	**6.40**	**+**	**+**	**+**	**+**	**+**	**+**	**+**	**+**	**+**	**+**	**+**	**+**	**+**	**+**	**+**
e-Recolnat	France	global; all	10.9 M	NA	0.16	−	+	+	−	−	−	−	+	−	+	+	+	−	−	+
Naturgucker	Germany	global; all	12.7 M	49.0 k	0.15	+	+	−	+	+	+	−	−	+	+	−	+	−	−	−
India Biodiv. Portal	India	ntnl; all	1.39 M	32.2 k	0.00	+	+	−	+	+	+	−	−	+	+	−	+	+	−	−
Biodiversity Maps	Ireland	ntnl; all	4.30 M	16.2 k	0.86	+	+	−	+	+	+	−	−	+	+	−	−	−	−	−
NLBIF	Netherlands	global; all	58.1 M	42.0 k	3.47	−	−	+	+	+	+	−	−	+	+	−	−	+	−	−
Bioportal	Netherlands	global; all	14.0 M	0.83 M	0.84	+	+	+	+	−	−	−	+	−	+	+	−	−	−	−
NBIC	Norway	ntnl; all	31.9 M	48.5 k	5.98	+	+	+	+	+	+	−	−	+	+	−	+	+	+	−
SABIF	South Africa	global; plants	1.42 M	0.11 k	0.02	−	+	+	+	+	0	−	+	−	+	−	−	+	−	−
Artportalen	Sweden	ntnl; all	73.6 M	34.3 k	7.19	−	−	+	+	+	+	+	−	+	+	−	+	+	−	+
Analysportalen	Sweden	global; all	83.7 M	NA	8.18	−	−	+	+	+	0	−	−	−	+	−	−	−	0	+
Dyntaxa	Sweden	ntnl; all	NA	NA		−	+	−	−	−	−	−	−	−	−	−	−	−	0	−
Bioatlas	Sweden	global; all	81.2 M	65.8 k	7.94	−	−	+	+	+	+	+	−	+	+	−	−	+	+	+
iDigBio	USA	global; all	0.12 G	NA	0.37	−	+	+	−	−	+	+	+	−	+	−	−	−	−	−
BISON	USA	global; all	0.47 G	NA	1.41	+	+	+	+	+	−	−	−	−	+	−	−	−	−	−
NHM Data Portal	Institutional	global; all	4.50 M	0.55 M		−	+	+	−	+	+	+	+	−	+	−	−	−	−	−

Content coverage refers to (principal) geographic and taxonomic focus of data treated (all = no taxonomic restriction; ntnl = national). Numbers of records and species in the data of the BDDIs were queried in late 2019 to 05/2020 and are therefore indicative rather than exactly comparable. Records per capita is the number of records divided by the national population of the data-sourcing area. A–G are data types covered by the BDDI: A = verbal descriptions; B = master taxonomy; C = collection specimens; D = opportunistic observations; E = systematic observations such as surveys, mapping; F = event-based observational data; G = DNA barcodes. 1–8 are services provided by the BDDI: 1 = data generation, such as digitisation of natural history collections; 2 = possibility for citizen science users to enter observations; 3 = collation & aggregation of data, i.e., gathering datasets from different sources to one repository and making them a single interoperable and reusable data mass; 4 = a collection management system (CMS); 5 = possibility for users to annotate records/identifications; 6 = Red List classifications or administrative statuses of species; 7 = red-listing tools; 8 = e-Lab services, such as dashboard and/or analysis functions for data visualisation and exploration. Marking: − = no; +  = yes; 0 = not applicable or data unavailable.

Specialisation may represent a reasonable division of labour at the macro level and be the only feasible way to advance service generation. However, in many countries, the mosaic pattern has been repeated at the national level (e.g., Germany, Netherlands, Sweden, Taiwan, UK, USA). When this happens, the benefits of narrowly defined missions may be lost, and the silo effect may hinder effective development. Fragmentation into separate service models, which could be integrated, runs the risk of creating counterproductive competition and redundancy, complicating the service landscape, impeding cross-sectoral and international cooperation, and frustrating funders. Here, we present the recently established Finnish Biodiversity Information Facility (FinBIF) to provide a best-practice model of a comprehensively integrated, cross-sectoral BDDI.

## Results

### The integrated service model of FinBIF

**Fig. 2:**
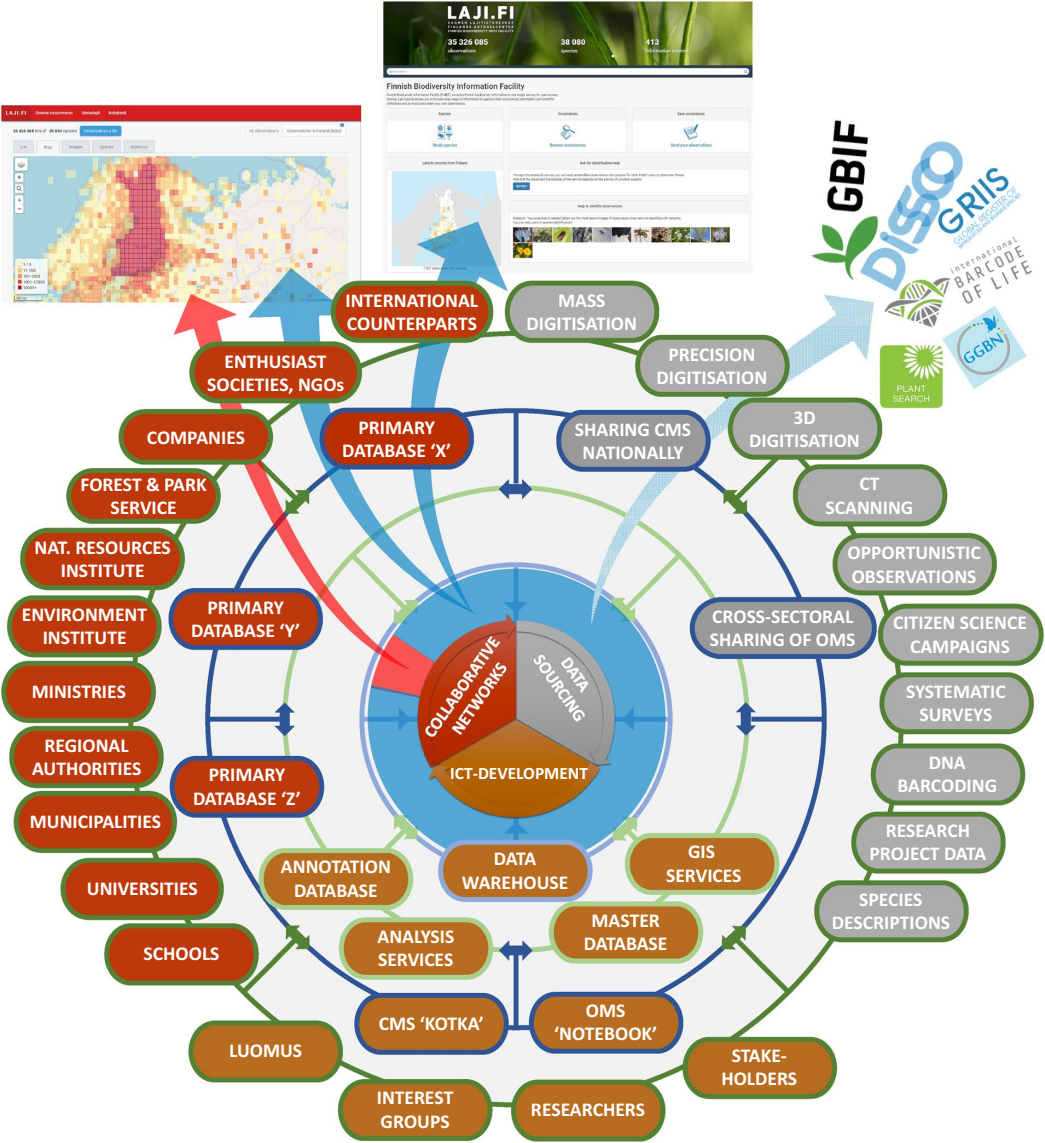
The structure of FinBIF. Data mobilisation (grey segment), ICT development (brown), and cross-sectoral networking (red) are integrated. Data providers, data-generating processes and collaborators (outermost, dark green circle) feed data and expertise to existing or FinBIF-made databases (dark blue). The data go through a standardisation and annotation process (light green) into the data warehouse to form one data mass controlled for use restrictions (blue sector = open, red = restricted-use data). The portal Laji.fi is the gateway to the harmonised open data. Restricted-use data go to authorities via a separate portal. Open data are also channelled to international systems.

FinBIF is like a scale model of the global biodiversity informatics landscape^5^ (for the *species* information part, disregarding the *ecosystem-level* and *policy* components). The service missions that are chiefly separate in global BDDIs are all incorporated into FinBIF (Fig. 1). Particularly, combining processes and services *generating* digital data with sourcing, collating, integrating and distributing *existing* digital data is rare (Table 1). FinBIF includes digitisation of collections, the development of data systems for collections and observations, and the construction of a national DNA barcode reference library (Fig. 2). Data flows are managed under a single IT architecture, services are delivered through the same on-line portal, collaboration coheres under a single umbrella concept, and development visions are presented to funders under one brand.

FinBIF’s service portal is available at https://laji.fi or https://species.fi (in Finnish, Swedish and English). Below we describe the data life cycle phases, data types, and services covered by FinBIF. Since FinBIF applies the Agile Development approach, the functionalities described here are improved almost continuously. Some of them are under construction at the time of writing, but most are operational.

#### Taxonomic data standardisation

FinBIF builds and maintains a taxonomic backbone for native species, which also serves as a national checklist^6^. It will be mapped to Catalogue of Life (CoL) enabling international distribution, once GBIF has adopted CoL as its taxonomic backbone, and CoL will then also provide the taxonomy for non-native taxa in FinBIF. Currently, 41,000 species are included (85% of the estimated total Finnish multicellular biota). The taxonomic database includes administrative attributes of species, such as threat, conservation or invasiveness status^7^.

#### Textual information and images

FinBIF’s species pages (e.g., https://laji.fi/en/taxon/MX.38815) include verbal descriptions of morphology and biology. Images from various sources are displayed.

#### Data generation: multi-technology digitisation of specimens

FinBIF coordinates national efforts of collection digitisation. It provides equipment, expertise, and R&D in digitisation, and avenues for data flow from collection-holding institutions to the common data portal. Technologies include conveyer-belt-based high-throughput imaging, high-resolution scanning and photography, focus stacking photography, and 3D scanning.

#### Data generation: DNA barcoding

FinBIF embraces FinBOL, the Finnish branch of the International Barcode of Life (iBOL), which supports the generation of DNA barcode data. The data are delivered to users through iBOL’s BOLD system but connected to species information at FinBIF’s portal (e.g., https://laji.fi/en/taxon/MX.204552).

#### Data sourcing: providing a collection management system (CMS)

The CMS ‘Kotka’, has been developed and provided for national use within the remit of FinBIF^8–10^. It is used by 11 Finnish natural history collections. The primary data held in Kotka are copied to FinBIF’s data warehouse and distributed through its portal.

#### Data sourcing: systematic observational data

FinBIF provides a primary data system for observational data, the observation management system (OMS) ‘Notebook’^11^. Notebook provides the data management platform for both continuous monitoring schemes^12^ and projects, like flying squirrel and invasive alien species (IAS) surveys (EU Life + projects) and Carbon Zero Dairy Farm biodiversity monitoring (Valio Ltd.).

#### Data sourcing: opportunistic observational data

Non-systematic observations can also be recorded in Notebook. Citizen science campaigns (e.g., Atlas of Finnish Fungi funded by a private foundation) and nature societies may collaborate with FinBIF and be provided with custom-made data entry forms. Additionally, FinBIF is a member of the iNaturalist Network, and supports a localised national portal, iNaturalist Finland. Finnish data sourced through it are copied to FinBIF’s data warehouse and integrated into the total data mass.

#### Data sourcing: private collections

Notebook enables private collection holders to use it as a CMS. It provides practical tools, such as a label-maker for specimens^9^.

#### Data sourcing: research data

Institutional digitalisation processes sometimes include active research data sourcing^13^, but national (and wider) initiatives have not had much direct involvement of data-producing research groups. FinBIF has created a direct dataflow between EarthCape Ltd’s database platform for biodiversity research^14^ and FinBIF’s data warehouse. EarthCape users are offered the option to share their data openly through this pipeline. FinBIF also seeks direct contact with research groups collecting significant sets of species occurrence data to offer services for data management, storage, and sharing.

#### Data aggregation, distribution and FAIRness

FinBIF stores copies of primary datasets in a bipartite data warehouse^15^: one part stores all data for restricted access by authorised users only, the other stores all open data and part of the restricted data with the use restrictions applied (spatial coarsening, postponed releases). Most data are fully open ( > 90%), but controlling for necessary restrictions (legal, contractual) requires a stringent data policy (https://laji.fi/en/about/2982). Despite the legal obligation on authorities not to release data that can harm endangered species, neither the species nor the potential forms of harm have been formally defined. Therefore, FinBIF catalysed a dialogue among national authorities and biodiversity specialists to produce a list of species whose data need restrictions on openness and rules by which the data are restricted (https://laji.fi/en/about/875).

The open data are distributed ‘as is’, and the restricted data after filtering by the stipulated restrictions, through FinBIF’s and GBIF’s portals (http://laji.fi; https://www.gbif.org/, respectively). Authorities can use all data through a parallel portal (https://viranomaiset.laji.fi/; access restricted). To allow researchers to acquire restricted data through a case-by-case evaluation by the data owners, FinBIF implements a semi-automated data request system^15^.

FinBIF has performed a self-assessment^15^ of how FAIR (Findable, Accessible, Interoperable, Reusable)^16^ its data distribution is. It is also being assessed for FAIR-uptake with several other digital repositories^17^ in an EOSC-Nordic project that supports participants in continuously approaching full FAIRness. The aim is to assess the aspects of FAIR maturity that will increase the trustworthiness of the repository. The datasets of FinBIF are scrutinized against FAIR using an automated assessment tool^18^ based on a FAIR maturity framework^19^. FinBIF aims for appropriate certification through the project.

#### Data quality management

Post-release quality enhancement is enabled through crowd sourcing. All entries can be annotated at FinBIF’s portal (identification etc.) and classified by their reliability. Users can also submit observations through a special form called ‘Which species?’ (https://laji.fi/en/vihko/MHL.9). Such observations are highlighted at the portal to allow enthusiasts to offer their expertise in identification. Datasets are classified also pre-release into three classes based on their presumed quality, which allows the user to filter data by perceived reliability. For instance, professional monitoring schemes and the digitisation of scientific specimens produce data of the highest quality.

#### Thematic data management services

FinBIF provides customised data management systems for some stakeholder organisations. The national hub on IAS (http://vieraslajit.fi), where information is provided and sightings can be reported, is governed by the state administration but built and maintained by FinBIF. It is integrated into FinBIF’s IT architecture and uses the same core data (e.g., taxonomy), although the hub itself has an independent design. FinBIF has also created a service for authorities and commissioned actors where information on IAS sites can be entered, and eradication actions and their results reported as part of EU reporting obligations.

Special functionalities were built in FinBIF to provide the latest national Red Listing^20^ with a data management platform enabling the evaluations and storing the information for subsequent retrieval and reporting^21^. FinBIF also built a public portal for browsing the information (https://punainenkirja.laji.fi/en).

#### Educational applications

FinBIF is integrated with the e-learning environment for species identification ‘Pinkka’ of the University of Helsinki (http://pinkka.helsinki.fi/pinkat). The services use the same taxonomy and feed content (textual information, images) into each other reciprocally. Additionally, primary schools have been encouraged to create digital herbaria and other species collections through a tailored service at FinBIF.

#### Data re-usability and usage support

Registered users will obtain a citable, unique HTTP-URI identifier^22,23^ for a downloaded data batch. The identifier is persistent and always resolves back to the original data and filtering criteria. Digital Object Identifiers (https://www.doi.org/) will also be provided in the near future.

‘Dashboard’ and ‘e-Lab’ are commonly used terms for functionalities that make it easier for users to understand the nature and composition of the data distributed, and to analyse them either online or after download. FinBIF provides a dashboard that factors the data by dataset, taxonomic group, and time and type of observation. E-Lab services are also within the scope of FinBIF, one example being an R programming language interface to the FinBIF application programming interface (API)^24^.

### User uptake of FinBIF

**Fig. 3:**
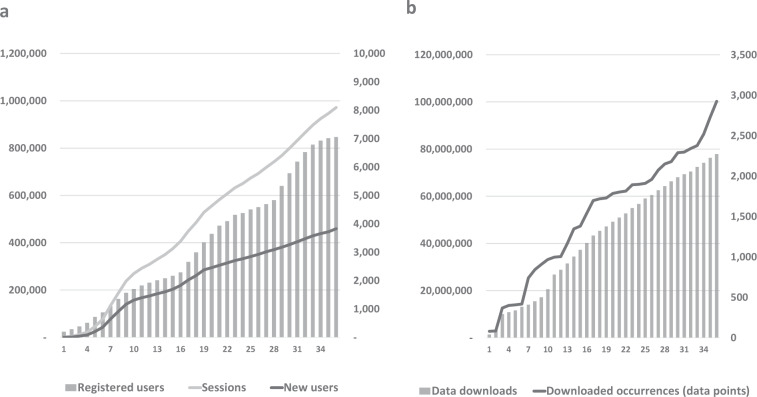
Use of FinBIF in 01/2017–12/2019 (the first three operational years) (**a**). Cumulative monthly number of new users and use sessions (left Y-axis) and registered users (right Y-axis) (**b**). Cumulative monthly number of data download events (right Y-axes) and total number of downloaded data points (left Y-axis).

In its first full operational year (2017), FinBIF attracted almost 175,000 new users and > 300,000 use sessions; 1,900 users registered to be able to down- or upload data (Fig. 3a). Downloads summed to 839 data batches containing > 34 million data points (Fig. 3b). After an initial lag phase, overall usage increased rapidly in the spring and summer months after which the growth decelerated. This pattern was repeated in 2018. In 2019, general usage grew steadily, but the growth in registered users accelerated and continued to show the seasonal pattern. Use sessions have increased faster than overall user numbers, indicating an increase in returning visitors. The rapid increase in registered users indicates the same; their number reached 7,000 before the end of 2019. Data downloading has increased more steadily at a slowly decreasing rate. The number of downloaded data points show an uneven growth due to great variation in the size of data batches. By the end of 2019, just over 100 × 10^6^ data points had been downloaded in total.

The background of the registered users of FinBIF varies, although the majority of the registrations come from private e-mail addresses. An institutional domain does not prove that the use is professional, or vice-versa, but the indication is that FinBIF attracts considerable interest from all sectors of society (Table 2).

**Table 2:** FinBIF’s registered users by category according to the domain of the e-mail address used for registration (12/2019). FinBIF is an open service and has had c. 450,000 users in total, but registration is required for entering data into FinBIF’s systems and for downloading of HTTP-URI-citable data batches.

User category	Number	Share of total (%)
Private citizen	5 556	79,5
Research and education	908	13,0
Private sector organisations	317	4,5
Bioeconomy enterprises – 0,7%	51	
Media – 0,2%	13	
Other companies – 2,8%	193	
NGOs – 0,9%	60	
State and municipal administration	207	3,0
**Total**	**6 988**	**100,0**

## Discussion

There are no universally agreed Key Performance Indicators for BDDIs. Numbers of datasets integrated, records offered, and species covered are commonly provided, but use statistics (numbers and types of users, amounts of data downloaded, numbers of citations in publications) are not readily available. In the absence of absolute measures allowing quantitative performance assessment, we find scenario analyses useful in evaluating the success of FinBIF: how would it have developed had alternative approaches been followed? Comparative data can support such analyses (Table 1). We acknowledge that our survey of BDDIs is not exhaustive. However, among those, FinBIF is uniquely inclusive, and we are not aware of any equally comprehensive BDDI.

FinBIF has rapidly mobilised and collated high volumes of data. While even higher numbers are demonstrated by some more narrowly specialised national BDDIs, these have operated for longer. FinBIF’s high data volume and diversity of datasets can be attributed to its inclusive coverage of the data life cycle and data types, and to wide cooperation across sectors of society.

Rapid user uptake and a diverse user base are strong indicators of the value of a BDDI. With less inclusiveness, FinBIF would have missed many potential users. In the research and education sector, users include researchers and collection curators, and teachers and students from all levels of education, because FinBIF offers vital data types and services for all these groups. Similarly, all levels of administration have a professional motivation to use FinBIF as it supports data management pertaining to endangered species and IAS. The high share of private users stems from providing a data management platform both for systematic species surveys, in which thousands of volunteers participate, and for citizen science projects and opportunistic observations by nature enthusiasts. Importantly, inclusion of data *recording* and data *sharing* services into the same BDDI results in a ‘snowball effect’ in which input of data enhances use, which in turn encourages further input.

Commonly acknowledged challenges for BDDIs are the taxonomic and temporal biases of the mobilised data^25^. Recent, born-digital observational data of popular taxa, notably birds, are overrepresented in comparison to historical data based on natural history collections, notably invertebrates. FinBIF compares favourably to other BDDIs in these respects, primarily due to the inclusion of an extensive programme for the digitisation of natural history collections right from the beginning.

In all, FinBIF’s performance in terms of output and usage has clearly benefitted from the chosen comprehensively integrated business model, but it is important to note also the model’s challenges. IT development becomes more demanding with more technical informatics requirements in, e.g., core data standardisation, data flow design, and data quality management. The diversity of user needs makes prioritising development tasks challenging. We believe that the only way to manage this complexity is to apply Agile Software Development. Numerous services that are now operational in FinBIF were not envisaged, when the vision of the BDDI was formulated in 2012. Furthermore, our experience is that an in-house IT team renders agile development considerably easier compared to outsourcing. One possibility to reduce technical requirements toward the development team is to apply modules of open-source software packages, such as that of the Living Atlases, which is a community created around the Atlas of Living Australia platform (https://living-atlases.gbif.org/). However, the downside is the dependence on the available components. For instance, in response to the needs of central stakeholders, FinBIF has produced a CMS for national use and a platform for the environmental administration for red list assessments as well as a restricted-use data request service, which are not available for Living Atlases. While maintaining the full service suite in-house places a higher resource burden on the FinBIF maintainers, the advantage is that all FinBIF’s components are interoperable from the start, increasing demand responsiveness and opening up a wider range of possibilities for service development over time.

Another important lesson learned is the need for active and extensive networking. Numerous cross-sectoral expert groups have been convened by FinBIF to support different aspects of its development. The service is thus the result of inclusive co-creation with data producers, owners and users from all sectors of society.

A non-technical but strategically pivotal benefit from an all-embracing business model is the possibility to tap into a variety of funding mechanisms. FinBIF has received considerable external funding from national and EU research infrastructure funding, allocated on the basis of scientific excellence, but sources unavailable to purely research-orientated initiatives have covered almost 40% of the total. These include governmental funds for administration enhancement, EU Life + funds for nature conservation, and private foundation grants for citizen science projects. Broad funder support testifies to the ‘saleability’ of FinBIF’s vision. Repeating the narrow specialisation seen in the fragmented global landscape of BDDIs^5^ on the national level may lead to the opposite, as recently seen in Sweden, where the national Research Council has pushed for the merger of previously separated BDDIs (https://tinyurl.com/rpwgog2).

We suggest that striving for the widest possible coverage of data types and data life cycle phases is best practice when building national BDDIs. However, some components of complete best-practice guidelines are still lacking, in particular solutions on long-term data archiving and on handling duplicate data points created through the integration of numerous primary datasets. Furthermore, the BDDI landscape is continuously evolving. FinBIF, like other national BDDIs, will have to implement new data standards to support data sharing with emerging infrastructures, such as the European Distributed System of Scientific Collections (DiSSCo; https://www.dissco.eu/).

Despite the remaining issues and the challenges in carrying through the comprehensively integrated model of FinBIF, we argue that its numerous benefits make it worthwhile. The landscape analyses and networking investments required for inclusiveness are manageable at the national level. Having one actor avoids redundancy in the development of IT components and services. An inclusive BDDI is straightforward to brand, making funder engagement more likely. Most importantly, making data FAIR^16^ is easier through a one-stop-shop, resulting in a user-friendly service as well as rapid and broad user uptake.

## Methods

### The contexts of FinBIF’s emergence and development

#### Build-up of impetus and consensus

A gradually strengthening drive from different sectors of society led to the eventual realisation of FinBIF as a comprehensively integrated BDDI. The earliest push came from a working group convened by the Ministry of Education and commissioned to analyse and plan the future of natural history museums (NHMs) in Finland. It identified as a goal to establish a national central NHM, one task of which would be to “act as an information centre maintaining an Automated Data Processing (ADP) based national register of collections”^26^. A subsequent working group commissioned to plan the central NHM concluded that one of the most urgent of the new nation-wide functions was “to create an ADP-based central registry to serve all Finnish natural history museums”^27^.

While the central NHM was established in 1988, the proposed inclusive, national database did not materialise, but separate actors (institutions, projects, research groups) developed a variety of information systems independently. When Finland joined GBIF in 2001, an increased enthusiasm on BD informatics emerged, but significant national funding for GBIF-related activities was not mobilised. However, the demand for national progress grew rapidly with the international development of BD informatics. A project on developing the efficiency of nature conservation made recommendations on IT development, and on collating and opening data through a national solution^28^. The National Red List^29^ included recommendations on developing databases for species observations. An action plan on developing species conservation recommended a separate project for overall development of biodiversity data management, which was already close to what FinBIF later came to be^30^.

In 2012, the Finnish Museum of natural History ‘Luomus’ successfully lobbied for a national, cross-sectoral meeting to discuss the way forward in building a national BDDI. This kick-off meeting was convened by the Ministry of the Environment on 7 September 2012. All major organisations that use, hold and/or produce biodiversity data, or fund such activities, were represented (Table 3). Subsequently, the urgency of establishing the national BDDI was re-emphasised in Finland’s Biodiversity Strategy and Action Plan^31^.

**Table 3:** The organisations represented at the meeting where the establishment of a national BDDI was agreed upon on 7 September 2012.

Stakeholder category (number)	Organisation
Universities (6)	Åbo Akademi University; University of Eastern Finland; University of Helsinki; University of Jyväskylä; University of Oulu; University of Turku
Ministries (5)	Ministry of Agriculture and Forestry; Ministry of Education and Culture; Ministry of the Environment; Ministry of Finance
Research Institutes (4)	Consortium of Natural Resources and Environment Research LYNET; Finnish Forest Research Institute METLA; Finnish Environment Institute SYKE; Finnish Game and Fisheries Research Institute RKTL
NGOs (2)	BirdLife Finland; Finnish Association for Nature Conservation
Other (4)	Åland Provincial Government; The Finnish Wildlife Agency; Natural Heritage Services of Metsähallitus; the national science funder Academy of Finland

#### Cross-sectoral co-creation, collaboration and funding

The host institution of FinBIF is the Finnish Museum of Natural History’Luomus’, which has led its development and the acquisition of funding, and coordinated the national and international cooperation. However, a wide national collaborative network has been an integral part of FinBIF from the start (Fig. 2), and many organisations from all sectors of society have participated in the development of FinBIF’s services and in data mobilisation. Luomus has convened seven advisory and co-creation groups in which c. 140 specialists have shared their expertise on a voluntary basis to advise policy and service development (Table 4). The wide collaborative network and the all-inclusive business model of FinBIF has helped in attracting funding for its development from many different sources with different funding criteria, such as national and EU-level research infrastructure funding, national funds for developing governance and administration, EU-funding for nature conservation actions, funds for Nordic collaboration in e-infrastructure development, private foundation grants to research and citizen science, and collaborative projects with private companies.

**Table 4:** The expert advisory groups convened by FinBIF for co-creation of content, services, and policies. CMS = collection management system; OMS = observations management system; API = application programming interface; GIS = geographical information system; INSPIRE = infrastructure for spatial information in Europe.

Group (with approximate number of participants; total c. 140)	Roles
Authorities (20)	Development of collaboration between and services for authorities; policies on data sharing
Research services (20)	Identification and prioritisation of services to be developed for research use.
Species information incl. taxonomy and names (20)	To find consensus on usage of names, taxonomies (incl. concepts), species descriptions etc.
Scientific collections and digitisation (12)	To map challenges and find solutions in collection management and digitisation; to steer the development of the CMS Kotka.
Observations, surveys and monitoring (40)	To source, mobilise and collate observational data; to contribute to criteria for restricting the use of sensitive data; to design the data quality control framework; to steer the development of the OMS Notebook.
Enterprise architecture (15)	To map data sources/information systems at each organisation represented; to describe the technical platforms and logical framework to be included into one common architecture documentation; to define needs and regulations in relation to the quality and usability of the data and services; to steer the building of APIs of FinBIF.
GIS, spatial and geographic data (12)	To ensure INSPIRE compatibility of FinBIF; to develop the services for public authorities to meet the requirements; to develop spatial services for all users; to enhance the collaboration among organisations managing geographic data.

### IT Architecture

FinBIF’s architecture has four key goals:

1. To harvest occurrence data from Finland and surrounding areas and share them as one interoperable data mass
2. To provide a customisable system for recording species observations
3. To provide a collection management system, and digitisation technologies and workflows for Finnish natural history collections
4. To compile, maintain, and share master data, including a taxonomic backbone of Finnish species.

These goals are accomplished by using Service-Oriented Architecture (SOA)^32^ and by providing over twenty individual background services that interact with each other, mostly using RESTful HTTP APIs (for explanation of REST, see^33^). These include: (1) A triplestore RDF database^34,35^ and an API for master data (taxonomy, schema vocabularies, people, collection metadata, image metadata, data download metadata) and collection specimen data; (2) a JavaScript Object Notation (JSON^36^) storage database and an API of primary occurrence data; (3) a Data Warehouse database, and an extract-transform-load (ETL) process and an API built on top of an HP Vertica database; (4) an Elasticsearch search engine (see https://www.elastic.co/what-is/elasticsearch) and an API for performance-critical access of primary occurrence data and taxonomy data; (5) multimedia services (images, audio storage and conversions); and (6) map services.

FinBIF is provided as a service to fulfil the needs of the Finnish biodiversity data community and to share Finnish data to all interested parties. The architecture and complete software of FinBIF cannot be easily adopted by other countries or institutions. However, FinBIF maintains several software libraries that are specifically designed to be reusable by anyone; such as the specimen label generator library, and the powerful form generation tools of the Notebook OMS. Data and services can be used by third party applications via FinBIF’s public API (https://api.laji.fi).

#### Occurrence data architecture

**Fig. 4:**
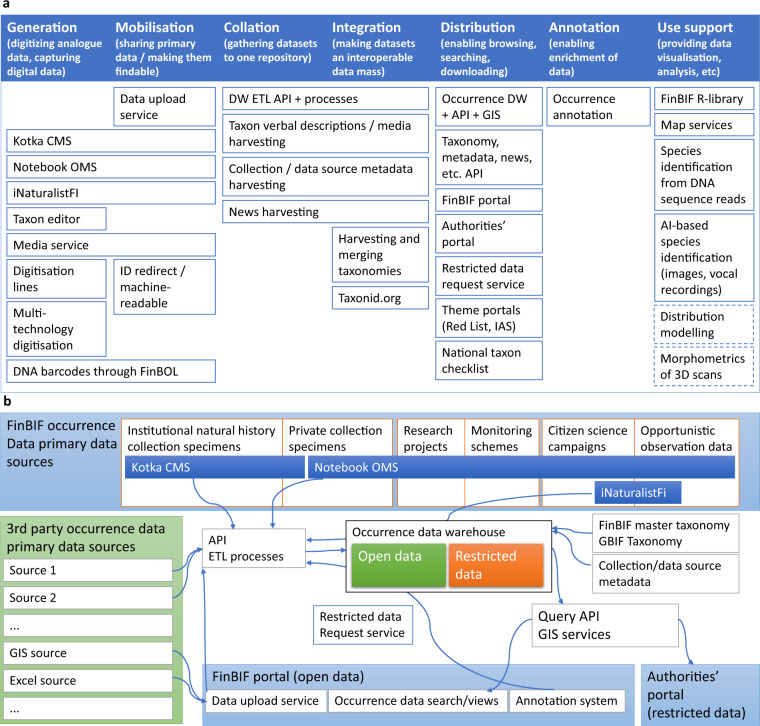
Services, processes and data flows of FinBIF (**a**). The relations of services and processes to data life cycle phases as depicted in Fig. 1. Dashed lines denote planned services (**b**). Occurrence data flow from FinBIF’s and external actors’ primary sources through FinBIF’s data warehouse to FinBIF’s portal. CMS = Collection Management System, OMS = Observations Management System, DW = data warehouse, ETL = Extract-Transform-Load, API = Application Programming Interface, GIS = Geographical Information System, IAS = Invasive Alien Species, AI = Artificial Intelligence.

FinBIF maintains systems for both primary and secondary occurrence data storage (Fig. 4). A primary data storage is a database or other type of storage where the data are maintained and used for the original purposes and use cases. A secondary data storage holds a copy of the primary data for distribution, analysis, and other derived purposes.

FinBIF has two IT systems for storing and maintaining primary occurrence data: the Kotka CMS for specimen data and the Notebook OMS for observational data (see below for descriptions). FinBIF also co-operates with iNaturalist and maintains iNaturalist Finland.

FinBIF’s Data Warehouse harvests occurrence data from primary data sources and stores them as secondary data. The data warehouse transforms primary occurrence data into a single interoperable data mass through various ETL processes. Currently, live updates are received from 34 different data sources using eight different ETL processes. Many more datasets have been loaded using a one-time process. For primary data sources that are not databases or IT systems (for example Excel, MapInfo files), a tool is provided for harmonizing the data into FinBIF format and uploading them to the data warehouse as secondary data. The primary data remain under the ownership and management of the source, where all changes and updates are made. However, FinBIF maintains an annotation system, which stores added information about the occurrence entries (as primary data), for example identifications and quality markings by taxon experts. These annotations are stamped on top of the original data in the data warehouse to provide an enriched version of the data.

Each dataset must have metadata that describe, e.g., the name, type and owner of the dataset. In the Data Warehouse, primary occurrence data, annotations, dataset metadata, the taxonomic backbone, and information on locations and people are linked to provide an enriched query service. FinBIF is a visible platform to make data FAIR^16^, thus increasing the prestige of the shared dataset, but the data sharer also gains through receiving quality feedback about the occurrences from the many taxon experts and volunteers that annotate the data in FinBIF. Based on the annotations, the data can be improved in the primary data source. To make data more interoperable, FinBIF encourages primary data source owners to harmonise their data with FinBIF-maintained master data, by using the same taxonomy and schema vocabularies.

The Data Warehouse has two sides: public and private. The public side contains open-access data, some of which have been coarsened because of the sensitivity of the species in question or because of a research embargo, as specified in FinBIF’s data policy (https://laji.fi/en/about/2982; https://laji.fi/en/about/875). In addition, individual observers may hide, e.g., the exact location of their occurrence or their name. The restricted-access side of the Data Warehouse contains uncoarsened data. Some data sources provide a limited version to the public side and a full version to the restricted side. Government officials can access the restricted-use, uncoarsened data via a separate authorities’ portal. Researchers and other users can do so by issuing a data request (see below). Maintaining two versions of the data has required developing unique designs. They do not reveal information about sensitive species, which might endanger their preservation, but still allow joining annotations made on the restricted side so these can be shown also on the public side. Occurrences that need to be coarsened are detached from their original concept and uploaded with a random delay to the public side to make it difficult to discover the exact location from accompanying occurrences. Apart from this delay, the private and public sides are automatically fully synchronised.

API and GIS services have been built on top of the Data Warehouse to allow open-access use of the data, both for the public and the restricted side. Most of the occurrence data from Finland go to GBIF via FinBIF. This data transfer is still being developed, and all datasets are not yet automatically copied to GBIF.

#### Master data management architecture

A triplestore is used in FinBIF for all small datasets, including taxon data. More specifically, the data are stored according to the RDF^35^ specification. An RDF Schema defines the allowed properties for each class. FinBIF’s triplestore^34^ implementation is an Oracle relational database with two tables (resource and statement), which provides the ability to do Structured Query Language (SQL) queries and updates. Doing small, atomic updates is easy, as only a small subset of the triplets can be updated instead of the entire data entity. Maintaining a complete record of history comes without much effort, as it can be done on an individual triplet level.

As an example, the FinBIF taxon data model – including adjacent classes such as publication, person, image, and threat assessments – consists of 260 properties. If the data model were stored in a normalized relational database, there would be an estimated 56 tables, which could be difficult to maintain. Thus, in FinBIF, non-relational database solutions are preferred.

#### Identifiers

FinBIF uses a persistent HTTP-URI identifier for all types of real-life and digital objects (specimens, occurrences, taxa, metadata, persons, organisations, information systems, etc.), as recommended by the World Wide Web Consortium^37^. The identifier takes the user to an ID redirect service, which redirects the user to a page that shows information about the object in human-readable format. For example, specimen identifiers redirect to information about the specimen and taxon identifiers to a page describing the taxon.

The redirect service can also provide machine-readable data about the object, if the user (client software) requests that using Accept headers. Supported formats vary based on data types, e.g., for specimens, the system can offer data in RDF + XML^38^ and JSON-LD (see https://json-ld.org/) formats using CETAF compliant vocabulary (CETAF Specimen Preview Profile CSPP^39^). This is also compatible with MIDS (Minimum Information about a Digital Specimen^40^).

If partner organisations do not provide HTTP-URI identifiers for their occurrences, FinBIF will use the persistent internal IDs of the data source to generate globally unique URI identifiers. DOI identifiers for data downloads and dataset metadata will be created in the near future.

### IT solutions in key services and processes

FinBIF runs numerous processes to provide a rich set of services (Fig. 4). The IT solutions applied in building key services and in enabling central processes are described below. The order of the descriptions follows the data life cycle phases identified in Fig. 1.

#### Kotka CMS

Kotka is one of the two primary data management systems of FinBIF. It is designed to fit the needs of different types of collections and can be further adapted when new needs arise.

Kotka differs in many ways from traditional CMS solutions. It applies simple and pragmatic approaches. This has helped it grow into a nationally used system despite limited development resources – on average less than one full-time equivalent developer. The aim is to improve collection management efficiency by providing practical tools. Kotka emphasises the quantity of digitised specimens over completeness of the data. It harmonises practices by bringing all types of collections under one system; the types currently covered include zoological, botanical, mycological and palaeontological museum collections, tissue and DNA samples, and botanic garden and microbial living collections.

Kotka stores data mostly in a denormalised free text format using a triplestore and a simple hierarchical data model. This allows greater flexibility of use and faster development compared to a normalized relational database. New data fields and structures can be added easily as needs arise. Kotka does some data validation, but quality control is seen as a continuous process and is mostly done after the data have been recorded into the system. The data model is loosely based on the Access to Biological Collection Data (ABCD) standard^41^, but has been adapted for practical needs.

Kotka is a web application. Data can be entered, edited, searched, and exported through a browser-based user interface (UI). However, most users prefer to enter new data in customizable MS-Excel templates, which support the hierarchical data model, and upload these to Kotka. Batch updates can also be done using Excel. Kotka stores all revisions of the data to avoid any data loss due to technical or human error.

Kotka supports designing and printing specimen labels^9^, annotations by external users, and handling accessions, loan transactions, and the Nagoya protocol^10^.

#### Notebook OMS

Notebook is the other primary data management system of FinBIF. It is a web solution for recording opportunistic as well as sampling-event-based species observations. It is being used for systematic monitoring schemes, various citizen science projects, and platforms for species enthusiasts.

Notebook’s main software component is LajiForm, which is the engine that renders a given JSON Schema into a web form. LajiForm is a separate, reusable module that is fully independent from other FinBIF systems. Notebook as a whole includes other features embedded in FinBIF, such as adding complex geographical shapes to observation documents, importing data from spreadsheets, and form templates.

All Notebook forms use FinBIF’s ontological schema in the JSON^36^ Schema format. Rendering user-friendly web forms based on a single schema is difficult, because the web form should be asking meaningful questions, instead of just rendering the schema fields according to the form description. Questions should be presented in an interactive manner. For instance, after drawing a geographical location on a map for a potential flying squirrel nesting tree, one would ask “did you see droppings at the nest?”, and answering “yes” would update the document to include a flying squirrel taxon identification with fields “breeding” and “record basis” filled in but not rendered to the form. A simpler form engine without a user interface (UI) customisation layer would just render the fields “taxon”, “breeding” and “record basis”, and the user would have no understanding why there are so many fields to fill in and how they relate to their work or study.

Some Notebook forms are complex, e.g., for experienced biology enthusiasts who need a form that is advanced, customisable, and compact. Some forms are simple, e.g., for elementary school children. To tackle this, LajiForm uses a separate schema for the UI that allows everything from simple customisation, such as defining widgets for fields, changing field order or customising field labels, to more complex customisation like transforming the schema object structure, defining conditions when certain fields are shown, or if updating a field should have an effect on other fields. All the functionality is split into a loosely coupled collection of components, which can be either used as standalone components or composed together in order to achieve more advanced customisation. The programming philosophy has drawn inspiration from functional programming, which has been helpful in writing isolated, composable functionality.

LajiForm is written with the JavaScript framework ‘React’. LajiForm is built on top of react-jsonschema-form (RJSF), which is an open source JSON schema web form library founded by Mozilla (see https://react-jsonschema-form.readthedocs.io/en/latest/). RJSF handles only simple customization, but it is very flexible in design and allows building extensions with features that are more powerful. Some features and design proposals were submitted to Mozilla – FinBIF is the largest outsider code contributor to RJSF, with a dozen pull requests merged.

#### iNaturalistFi

iNaturalist (https://www.inaturalist.org/) is an international observation and citizen science application and platform. The iNaturalist Network is a collection of websites that are localized to national use in c. 10 different countries. FinBIF supports the Finnish network site iNaturalist Finland through translations, instructions, communication, moderation, and user support.

Finnish iNaturalist data are automatically synchronized weekly to FinBIF’s data warehouse, where they are available for local use. iNaturalist observations are linked to the observer’s own account in the FinBIF portal, if they have linked their iNaturalist and FinBIF accounts. Both features are important in encouraging observers to use iNaturalist, and to allow it to work seamlessly with other FinBIF services.

#### Taxon Editor

FinBIF has developed its own taxon database, ‘Taxon Editor’. It allows taxon specialists to maintain their own, expert-validated view of Finnish species. The aggregation of these is used as a backbone taxonomy for all FinBIF services, and the national checklist of Finnish taxa is extracted from it (see https://laji.fi/en/theme/checklist). Each taxon is given a globally unique persistent HTTP-URI identifier, which refers to the taxon concept, not to the name. The identifier does not change if the taxon concept remains unchanged. Compatibility with checklists from other countries is sought by linking taxon concepts as Linked Data.

The taxon specialists (currently c. 60) maintain the taxon data using a web application. All changes made go live every night. The nightly update interval allows the specialists a grace period to make their changes. To maintain the integrity of critical data, such as lists of protected species, limitations to what the specialists can do have been imposed. Changes to critical data are carried out by an administrator.

Taxon Editor has special features for linking observations to the taxonomy. These include hidden species aggregates and tools to override how a certain name used in observations is linked to the taxonomy. Misapplied names, however, remain an unresolved problem. Most observations are still recorded using plain names, but it is possible for the observer to pick a taxon concept instead, which is the most precise way. When data are published through the FinBIF portal from other information systems, the data providers can link their observations to FinBIF’s taxon concepts by providing the concept’s identifier. The ability to use taxon concepts as a basis of observations means the concepts have to be maintained over time, a task that may become arduous in the future. For further description of the functionalities of Taxon Editor, see^7^.

Taxon Editor is also used in Red List assessments^21^. The threat assessment is carried out using the criteria of the International Union for Conservation of Nature (IUCN). FinBIF offers a documentation tool and an archive for the assessment, which is based on the national checklist of Finnish taxa. Information about previous assessments is available in the tool, and the assessor can copy and confirm, e.g., area of occupancy, extent of occurrence, generation length and habitat preferences of a species from the previous assessment. The service offers the possibility to add notes to most of the fields separately and commenting on the assessments by other authorised users. In line with the IUCN instructions, the tool automatically chooses the criteria leading to the highest possible threat category of criteria filled out for the species, although the assessor confirms the final evaluation. In several fields, the tool automatically checks the validity of values entered, e.g., criteria, threat category, length of observation period, causes of threat, and current threat factors. The tool includes necessary fields for backcasting the categories of previous assessments to count the Red List Index^42^. There is also a possibility to do regional threat assessments. Data are stored to the triplestore^34^, which archives the history of all changes.

#### Media service

The FinBIF media service currently supports receiving, transforming, storing and serving images and audio. For images, the original media is stored, and the service generates a smaller JPEG version to be used in the web. The service also generates different sized thumbnails. Only a handpicked set of Exchangeable image file format (Exif^43^) metadata is kept, so that the metadata would not leak location information about sensitive species occurrences. For audio, wav and mp3 formats are supported. The original file is processed (cleaned) to prevent any malicious content. Mp3, wav and a spectrogram are generated and stored. Support for the International Image Interoperability Framework (IIIF; https://iiif.io/about/) standard is under planning.

#### Data upload service

Many occurrence datasets are not yet maintained in modern IT systems or databases, which could use an API to transfer data. FinBIF is able to receive data from Excel and GIS systems as secondary data to its data warehouse. First, metadata are generated about the dataset, and selected users are given access to upload data to that dataset. Then the owner of the data must transform the data to a row/column-based table, i.e., MS-Excel or tab-separated value (TSV) file. Each row has one occurrence and must have an ID that is unique to that dataset. The dataset owner then proceeds to upload the row/column-based file using a Web UI, in which the owner maps the fields and values of their data to FinBIF schema fields and values. Updates are done by re-uploading the entire dataset or only a part of it. Deletions are handled by a specific column that annotates that occurrence as deleted.

#### Taxonomy backend

The taxonomy backend transfers the data created in Taxon Editor to an Elasticsearch search engine on a nightly basis. FinBIF’s API is built on top of the data in Elasticsearch. GBIF’s taxonomic backbone is currently being integrated into FinBIF’s taxonomy for taxa with occurrences but no taxonomy in FinBIF. This allows FinBIF users to browse a taxonomy that is a combination of the FinBIF and GBIF taxonomies. The taxonomy backend could harvest descriptions and images from other sources, but currently these functionalities are disabled because of data quality problems.

#### Collection metadata backend

Collection and dataset metadata are maintained primarily using the Kotka CMS, but metadata are also harvested from partner organisations’ metadatabases. The metadata of a collection contain the taxonomic, geographic and temporal coverage of the dataset, as well as information about its quality using a three-level grading: (1) Professional; (2) Expert hobbyist / expert curated; (3) Citizen science / mostly non-curated. Occurrence data can be filtered in the FinBIF data warehouse based on these levels. Each occurrence also has its occurrence-specific quality grading, which is different from the dataset grading. The collection metadata define, e.g., the people who handle data requests, which are done in FinBIF’s restricted data request service.

#### Taxonid.org

Finland and Sweden are piloting, with a subset of taxonomic groups, to connect national checklists using Linked Open Data standards^44^ and agreed vocabularies. By using HTTP-URI as globally unique, persistent identifiers for taxon concepts^45^, the service provides both human-readable (Hypertext Markup Language, HTML) and machine-readable (Extensible Markup Language, XML) responses for client requests via a central checklist (http://taxonid.org/). Future steps include linking the national lists with the global reference checklist developed by Catalogue of Life (https://www.catalogueoflife.org/). Currently the service includes only taxonomic information, but the eventual goal is to share information also on genetics, images, and traits, as well as on conservation status and observations, in a standardised way. The work was part of the DeepDive project which was funded by the Nordic e-Infrastructure Collaboration (https://neic.no/deepdive/). The vision is to establish a regional infrastructure network consisting of Nordic and Baltic data centres and information systems, and to provide seamlessly operating regional data services, tools, and virtual laboratories.

#### Application programming interface (API)

A fundamental goal of FinBIF is to provide all data in machine-readable formats, so that the data and services can be used by third party applications. This can be accomplished using FinBIF’s public API (https://api.laji.fi). It provides access to all data available in FinBIF. The FinBIF portal is built using solely the public API, which should ensure the API is robust, of high performance, reliable and easy to use.

#### GIS services

FinBIF aims to share occurrence data in GIS formats (WFS, WMS; see https://www.ogc.org/standards/). These services are currently under construction.

#### Restricted data request service

The restricted-use data that FinBIF harbours play a crucial role in, e.g., land-use decisions and conservation. To make these data findable, and conditionally accessible, through the public portal, a Restricted-use Data Request Service (RDRS) has been employed. It allows any user with a valid justification to request access to restricted-use datasets. After selecting the required compilation of data, the user submits a standardised form with the required information. The often multiple data owners receive a notification and a request to log in to the data owners’ section of the portal to scrutinise the request. The owners may discuss the request privately among themselves to facilitate decisions. In case of discrepancy by separate owners, the user who submitted the request may still download the released part of the data.

#### Occurrence annotation

FinBIF maintains an annotation system, which allows adding information to all occurrence records in the data warehouse. Any registered user can add comments and mark occurrences as needing verification. Trusted users can be given an expert annotator status, which allows them to have more effect on how the occurrences are shown. Experts can change the identification (taxon) that is displayed by default, grade occurrences based on their quality (verified, unreliable, erroneous), and override other annotations. However, annotations never change the original occurrence, which is always kept available. Data owners can be notified about annotations regarding their data, so that they can check and correct possible errors in the primary data source.

#### R-library

FinBIF provides an R programming language interface to the FinBIF API. The FinBIF R package makes the publicly available data in FinBIF accessible from within R. Biodiversity information is available on taxonomy and taxon occurrence. Occurrence data can be filtered by taxon, time, location and other variables. The data accessed are conveniently preformatted for subsequent analyses. Documentation and download can be found at https://luomus.github.io/finbif/.

#### Map services

FinBIF has built a JavaScript map service on top of a popular Leaflet library. It provides import and export in various formats, support for the Finnish national coordinate systems and a legacy coordinate grid layout, which is still widely used as a basis of monitoring schemes, though no longer officially supported by Finland’s geographical authorities. The tool provides a rich collection of national and international map layers that are useful in reporting and evaluating occurrence data. The tool also allows calculating lengths and areas as well as drawing complex geographical shapes (e.g. survey polygons and polylines with buffer areas).

#### AI-based species identification

FinBIF cooperates with projects that utilise machine learning in species identification and data classification. They involve creating (semi-)automated identification pipelines for Finnish fungus species from images, and bat and bird species from audio recordings. FinBIF has provided images of fungi, is building a crowd-sourcing platform on which bird experts can produce training material, and aims to implement the Animal Sound Identifier software^46^ for building on-line identification services.

#### User authentication

FinBIF provides an authentication and authorisation service that can be used also by third party applications and websites. Separate authentication flows exist for websites and native applications. Users can create one FinBIF account per e-mail address and associate multiple authentication methods and other user identities to their account. For example, users can login with Google or Facebook credentials and associate their iNaturalist, Finnish Wildlife Agency and Finnish Bird Ringing user identities to their FinBIF account. This enables users to see the occurrence data they have entered into various systems as their own occurrences in the FinBIF portal.

#### Servers, service continuity, backup and disaster recovery

FinBIF uses services provided by CSC, the Finnish IT centre for science (https://www.csc.fi) and the University of Helsinki IT department. Both provide FinBIF several OpenStack (https://www.openstack.org/) based virtual servers and an OpenShift (https://www.openshift.com/) cloud platform. FinBIF is not committed to any certain Service Level Agreement (SLA), but its availability is above 95%. All primary data gathered by FinBIF are professionally backed up by the University of Helsinki. CSC IDA (https://www.fairdata.fi/en/ida/) is used to archive larger datasets, such as specimen images. The CSC digital preservation service (https://www.fairdata.fi/en/fairdata-pas/) will be used for long-term archiving.

### Development methodologies

#### Agile development

The FinBIF in-house ICT team uses a three-level development process. The first level provides capacity to do long-term planning. Epic level requests (entirely new parts of the infrastructure, inclusion of new data sources, new monitoring schemes) are compiled, prioritised and to some degree scheduled using a Trello board. The aim is to open this board for all stakeholders and the public, so that interested parties can track progress and focus of development. This board is maintained in biweekly sessions.

The second level is used to direct current focus. A combination of Kanban (see https://www.atlassian.com/agile/kanban) and Scrum^47^ methodologies is used. Every two weeks the status of ongoing epics is checked, and the epics for the next two weeks are decided. Unlike in Scrum, there is no attempt to estimate or set goals for the sprints. Rather, a Kanban-like free-flowing system is used, where things take as long as they take.

The third level is the implementation process for individual epics. Like in Scrum, each epic has a named product owner (PO). The PO communicates with the stakeholders and defines user stories. The development team turns the user stories into smaller tasks, which are maintained in a Pivotal tracker backlog (www.pivotaltracker.com).

It is hoped that, in the future, developers from partner organisations could be attracted to participate in the development process.

### Data acquisition for this paper

#### Comparative data for other BDDIs

The data shown in Table 1 and supporting the drawing of Fig. 1 were acquired by searching for information on the public internet portals of the infrastructures between July 2019 and March 2020. The data were then sent to the representatives of the infrastructures for checking and possible corrections. Altogether 27 infrastructures (eight global and 19 national or institutional) were contacted, and answers were received from 23 (six global and 16 national or institutional) infrastructures. The following information was gathered.

**What is:**

- The taxonomic coverage of your infra?
- Current number of records?
- Number of species you have information on?

**Does your infrastructure share the following types of data and information:**

- Natural history collection data?
- Opportunistic observation data?
- Observation data collected in systematic monitoring schemes?
- Verbal descriptions of species?
- Standardized taxonomic core data?
- DNA-barcodes (or links to BOLD database)?

**Does your infrastructure provide the following kinds of user services:**

- Possibility to enter event-based data, meaning several species observations linked together?
- A Collection Management System?
- IUCN red-listing tools?
- Red-list classifications or administrative statuses of species?
- e-lab services? What kind of?

**Is the infrastructure designed to enable:**

- Data generation, such as digitization of natural history specimens?
- Collation / aggregation of data?
- Entering observations by citizen science users?
- Annotation of records/identifications?

#### FinBIF’s user data

Total numbers of users and use sessions were acquired from Google Analytics (https://analytics.google.com) for the website laji.fi in its entirety. To determine which traffic belongs to which user, Google Analytics sends a unique identifier associated with each user with each hit. This is accomplished via a Client ID, a unique, randomly generated string that gets stored in the browser’s cookies, so subsequent visits to the same site can be associated with the same user. Using cookies allows identifying unique users across browsing sessions, but it cannot identify unique users across different browsers or devices. Hence, the user numbers presented here are slight overestimates of true numbers of different people using the service. The use sessions are total number of sessions within the given range of dates. A session is the period during which a user is actively engaged with the website. All usage is associated with a session.

The number of registered users comes from FinBIF’s own registry. Until the end of 2019, users had to register at the service to be able to download data from laji.fi to their own device. Subsequently, the requirement has been relaxed so that registration is required only to obtain a citable persistent unique HTTP-URI identifier for a downloaded data batch, but non-citeable so-called light downloads can be done without registration. Registration is also required to be able to record data to FinBIF’s primary data management systems and to annotate records at https://laji.fi/.

Numbers of data downloads and downloaded data points are logged by FinBIF itself. As of the beginning of 2020, the numbers reported in Fig. 3b include light downloads.

## Data Availability

Data generated or analysed during this study are included in this published article as such (Table 1) or as a summary only to avoid disclosure of personal information (Table 2). Data summarised in Fig. 3 are available from the corresponding author or from helpdesk@laji.fi on reasonable request. The data service described distributes data at the address https://laji.fi/ (https://species.fi/ for starting page in English) under the terms specified at https://laji.fi/en/about/2982.

## Code availability

All of FinBIF’s code is open source and available primarily in GitHub (https://github.com/luomus), though partly in Bitbucket (https://bitbucket.org/luomus/). The project is covered by the MIT license (https://opensource.org/licenses/MIT).
